# Characterization of ExeM, an Extracellular Nuclease of *Shewanella oneidensis* MR-1

**DOI:** 10.3389/fmicb.2018.01761

**Published:** 2018-08-03

**Authors:** Lucas Binnenkade, Maximilian Kreienbaum, Kai M. Thormann

**Affiliations:** Institute for Microbiology and Molecular Biology, Justus Liebig University Giessen, Giessen, Germany

**Keywords:** DNA, biofilm, membrane, endonuclease, *Shewanella*

## Abstract

Bacterial extracellular nucleases have multiple functions in processes as diverse as nutrient acquisition, natural transformation, biofilm formation, or defense against neutrophil extracellular traps (NETs). Here we explored the properties of ExeM in *Shewanella oneidensis* MR-1, an extracellular nuclease, which is widely conserved among species of *Shewanella, Vibrio, Aeromonas*, and others. In *S. oneidensis*, ExeM is crucial for normal biofilm formation. *In vitro* activity measurements on heterologously produced ExeM revealed that this enzyme is a sugar-unspecific endonuclease, which requires Ca^2+^ and Mg^2+^/Mn^2+^ as co-factors for full activity. ExeM was almost exclusively localized to the cytoplasmic membrane fraction, even when a putative C-terminal membrane anchor was deleted. In contrast, ExeM was not detected in medium supernatants. Based on the results we hypothesize that ExeM predominantly interacts with DNA in close proximity to the cell, e.g., to promote biofilm formation and defense against NETs, or to control uptake of DNA.

## Introduction

Extracellular DNA ubiquitously occurs in terrestrial, marine and fresh-water habitats as the product of passive or active cell lysis or active DNA transport (Vlassov et al., 2007; Ibánez de Aldecoa et al., 2017). The concentration varies substantially between various environments and was demonstrated to reach up to 20 mg g^-1^ in activated sludge and 0.31 g of total DNA/m^2^ in the top cm of deep-sea sediments. More than 90% out of these 0.31 g/m^2^ are thought to be extracellular DNA (Palmgren and Nielsen, 1996; Niemeyer and Gessler, 2002; Dell’Anno and Danovaro, 2005). Thus, extracellular DNA affects bacteria in many different settings and environments. Bacteria were demonstrated to exploit DNA as a source of carbon, nitrogen, and phosphorus. In deep-sea ecosystem functioning extracellular DNA was even found to play a key role in the marine phosphorus cycles (Dell’Anno and Danovaro, 2005; Pinchuk et al., 2008; Mulcahy et al., 2010; Seper et al., 2011; Ibánez de Aldecoa et al., 2017). Extracellular DNA represents an important source for an intra- and interspecies exchange and spread of genetic information by horizontal gene transfer (Molin and Tolker-Nielsen, 2003). In addition, DNA has long been recognized as a highly common and, many times, functionally and structurally important component of the extracellular matrix of bacterial biofilms (reviewed in Flemming and Wingender, 2010; Dragos and Kovacs, 2017).

Given the abundance and various important functions of extracellular DNA, bacteria require appropriate means to degrade and modulate this molecule accordingly. To this end, many bacteria produce extracellular nucleases, which may either be anchored to the cell envelope or completely released into the extracellular space. During biofilm formation, the activity of such nucleases was demonstrated to balance the extent of biofilm formation by promoting biofilm dispersal in various species such as *Staphylococcus, Neisseria, Haemophilus, Vibrio, Shewanella, Ralstonia*, and others (Mann et al., 2009; Gödeke et al., 2011a; Kiedrowski et al., 2011; Seper et al., 2011; Steichen et al., 2011; Beenken et al., 2012; Kiedrowski et al., 2014; Minh Tran et al., 2016). Extracellular nucleolytic activity prevents DNA to accumulate to levels toxic to the cell, an effect that is due to the cation-chelating properties of DNA (Mulcahy et al., 2008; Heun et al., 2012). In addition, extracellular nucleases are crucial for utilizing DNA as a nutrient (Pinchuk et al., 2008; Mulcahy et al., 2010; Gödeke et al., 2011a; Heun et al., 2012). Moreover, they affect transformation activity and horizontal gene transfer by either degrading extracellular DNA from the environment to prevent entry into the cell (Focareta and Manning, 1987; Wu et al., 2001; Blokesch and Schoolnik, 2008) or as component of the natural DNA-uptake machinery (Berge et al., 2002).

In many pathogenic bacteria, another important function of extracellular nucleases is to promote the degradation of and escape from neutrophil extracellular traps (NETs). Such NETs consist of a DNA-matrix as a scaffold for an arsenal of antimicrobial proteins to degrade potential virulence factors and to immobilize and kill invading bacteria (Brinkmann et al., 2004; Halverson et al., 2015). A role of extracellular nucleases in evading NETs has been shown for a wide range of bacterial pathogens including *Vibrio cholerae, Streptococcus* sp., *Staphylococcus aureus, Prevotella intermedia*, and *Mycoplasma pneumoniae* (Beiter et al., 2006; Buchanan et al., 2006; Berends et al., 2010; Seper et al., 2013; Doke et al., 2017; Liu et al., 2017). Recent work has shown a similar role of extracellular nucleolytic activity for the plant pathogen *Ralstonia solanacearum* to degrade DNA deposited into the soil by root border cells to trap pathogens (Hawes et al., 2011; Tran et al., 2016).

Taken together, extracellular nucleases play a central role for the modification and/or degradation of eDNA in microbial biofilms and microbial communities and exhibit diverse functions with medical relevance, such as natural transformation, degradation of DNA in NETs, and induction of biofilm dispersal for biofilm control. However, the underlying molecular and regulatory mechanisms are still poorly understood and remain to be elucidated in more detail. In this study we further characterized the extracellular nuclease ExeM from *Shewanella oneidensi*s MR-1.

Generally, bacteria of the genus *Shewanella* are facultatively anaerobic gammaproteobacteria, which are characterized by their ability to use an enormous arsenal of external electron acceptors for respiration, a high tolerance for different sodium chloride concentrations and the capability to grow in a wide range of temperatures (reviewed in Hau and Gralnick, 2007; Fredrickson et al., 2008). Hence, *Shewanella* sp. have been isolated from a wide range of habitats from fresh and salt water sediments to rotten fish, and some *Shewanella* species have been identified as commensal human pathogens (Janda and Abbott, 2014). *S. oneidensis* has emerged as a model species for *Shewanella* physiology and biofilm formation. Previous work provided evidence that extracellular DNA is an important structural component of *S. oneidensis* biofilms and is produced by prophage-induced lysis in particular during cell-surface attachment due to iron-induced intracellular stress (Gödeke et al., 2011b, 2012; Binnenkade et al., 2014). In addition, this species is capable of using DNA as sole source of carbon, nitrogen, and phosphorus (Pinchuk et al., 2008). *S. oneidensis* MR-1 produces three extracellular nucleases, EndA, ExeS, and ExeM (Gödeke et al., 2011a; Heun et al., 2012). While EndA primarily degrades DNA in culture supernatants and is mainly required to use DNA as source of phosphorus, ExeS, and ExeM contribute only little to the nucleolytic activity in the supernatant. In contrast, ExeM, which was proposed to be associated with the cell envelope, strongly affects biofilm formation and cellular detachment dynamics (Gödeke et al., 2011a; Heun et al., 2012). The putative ortholog of ExeM in *V. cholerae*, Xds, was, similarly, shown to be involved in normal biofilm formation, nutrient acquisition and escape from NETs (Seper et al., 2011, 2013), strongly indicating an important role of ExeM/Xds for various cellular processes. However, rather little is known about the activity and localization of these nucleases. Here we performed a deeper characterization of ExeM to further elucidate its potential functions in DNA degradation.

## Materials and Methods

### Strains and Growth Conditions

Supplementary Table 2 shows all bacterial strains used in this study. Routinely, *S. oneidensis* and *E. coli* were cultivated in LB medium at 30 and 37°C, respectively. Agar was added to a final concentration of 1.5% (w/v) for solidification purposes. If needed, 2,6-diaminopimelic acid (300 μM), ampicillin (100 μg ml^-1^) and/or kanamycin (50 μg ml^-1^) were added. For biofilm assays of *S. oneidensis* MR-1, cells were grown in LM medium containing 0.5 M lactate (Paulick et al., 2009).

### Strain Constructions

Standard protocols (Sambrook et al., 1989) were applied for general DNA manipulations using kits for preparation and purification of DNA (VWR International GmbH, Darmstadt, Germany) and enzymes (Fermentas, Sankt Leon-Rot, Germany) accordingly. Plasmids (Supplementary Table 3) were introduced into *S. oneidensis* MR-1 by conjugation from *E. coli* WM3064. For markerless in-frame deletions sequential homologous recombination was applied using suicide vector pNPTS-138-R6K as previously described (Lassak et al., 2010). To construct vectors, either Gibson assembly (Gibson et al., 2009) or standard restriction/ligation methods were used. The oligonucleotides used for the required PCRs are shown in Supplementary Table 4.

### Cultivation and Quantification of Biofilms

Static biofilm assays were performed essentially as previously described (Thormann et al., 2004). Briefly, 170 μl LM medium per well on a 96 well plate (Sarstedt, Nümbrecht) was inoculated with 5 μl of a *S. oneidensis* overnight culture (8 wells per strain) and incubated at 30°C for 24–48 h. Optical density at 600 nm was determined before adding 10 μl 0.5% crystal violet to each well. After incubation for 10 min at RT the whole supernatant was discarded and 200 μl were added to wash the cells. Again, the supernatant was discarded and finally 200 μl 96% EtOH (w/v) were added and incubated for 5 min at RT before determining the absorption at 580 nm. To obtain relative biofilm formation the degree of surface attachment was normalized to that of the wild-type. At least three independent experiments were carried out.

### Enrichment of ExeM

**FIGURE 1:**
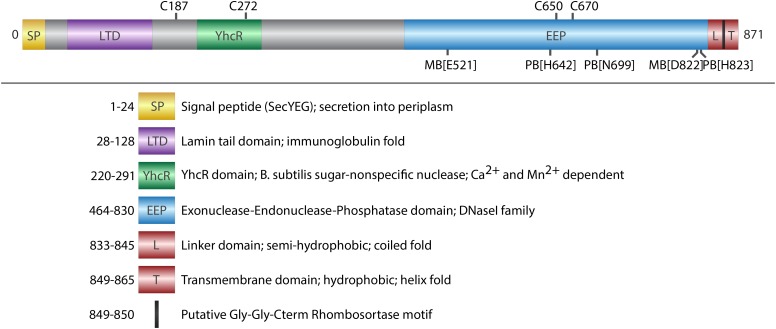
Domain architecture of ExeM. Schematic illustration of conserved domains identified by sequence comparison (BLASTP). Cysteine residues that are potentially involved in disulfide bond formation are indicated on the upper side. Putative metal binding sites (MB) and putative phosphate binding sites (PB) are indicated on the lower site. Deletions of the domains were designed in a way that the exact amino acids of the respective domain were deleted, except deletion of L-TM, where amino acids 833–869 were deleted.

For construction of over expression plasmids, vector pMal-P2X (NEB) was altered by exchanging the Factor Xa protease cleavage site for a TEV protease cleavage site, resulting in plasmid pMal-P2-TEV. Then, plasmids encoding ExeM, ExeM-ΔLTD (ExeM lacking the N-terminal LTD domain; **Figure 1**), ExeM-ΔYhcR (ExeM lacking the YhcR nuclease domain; **Figure 1**) and ExeM-ΔEEP (ExeM lacking the C-terminal EEP nuclease domain; **Figure 1**) were constructed by amplifying the appropriate gene regions without both the N-terminal signal sequence and the C-terminal hydrophobic regions and cloning it into pMal-P2-TEV. This resulted in in-frame fusions to *malE*, encoding the maltose binding protein (MBP) and targeting of the fusion protein to the periplasm. *E. coli* BL 21 Star (DE3) was transformed with the resulting vectors. 400 ml SOB medium containing 0.2% (w/v) glucose were inoculated with an overnight culture in LB (containing 50 μg ml^-1^ ampicillin) of the resulting strains. Cells were grown at 37°C to an OD_600_ of 0.5, rapidly cooled down on ice and IPTG was added to a final concentration of 0.3 mM. After incubating the cultures for 4 h at 25°C, the cells were harvested by centrifugation, frozen in liquid nitrogen and subsequently stored at -20°C. Cell pellets were lysed by resuspending them in 30 ml ice-cold 1× PBS buffer containing 0.5 mM AEBSF-hydrochloride (Carl Roth GmbH + Co. KG, Germany) and subsequently passing them through a cooled “French Press” three times. Centrifugation at 8,000 *g* for 10 min at 4°C and subsequent ultracentrifugation at 30,000 *g* at 4°C were used to remove unbroken cells and insoluble cell debris, respectively. At 4°C, the solution was added to a column containing 5 ml amylose resin and eluted in 1.5 ml fractions by adding column buffer (20 mM Tris-HCl, 200 mM NaCl, 10 mM maltose). The elution fractions were then analyzed via SDS-PAGE and pooled if containing high levels of protein. Five hundred microliter of the sample were further purified by size-exclusion chromatography (Superdex 200 HR 10/30, GE healthcare) using Buffer A (20 mM Tris-HCL, 200 mM NaCl, pH 7) for isocratic elution. Protein concentration was measured with a spectrophotometer (NANODROP 1000, Thermo Fisher Scientific). After another analysis by SDS-PAGE, 30% (v/v) glycerol was added to the samples, which were then frozen in liquid nitrogen and stored at -20°C.

### *In vivo* and *in vitro* DNA Degradation Assays

#### Nuclease Activity in Culture Supernatants

Qualitative nuclease assays in medium supernatants were conducted essentially as previously described (Gödeke et al., 2011a). Appropriate cells from an overnight LB preculture were incubated in fresh medium at an OD_600_ of 0.05 and were grown to an OD_600_ of 1.5. 230 μl aliquots of cell-free filter-sterilized supernatant were mixed with an appropriate nucleic acid sample at a final concentration of 5 μg ml^-1^. The samples were incubated at 30°C, aliquots were removed at regular intervals and the integrity of the DNA was analyzed by agarose gel electrophoresis. The assay was repeated in at least two independent experiments.

#### Nuclease Activity of Washed Cells

For a comparison of nuclease activity in culture supernatants and washed cells, cultures were grown to an OD_600_ of 1.5. In order to inhibit further protein synthesis, chloramphenicol was added to a final concentration of 30 μg ml^-1^ and cultures were incubated for 20 min at 30°C. To determine the nuclease activity of washed cells, 230 μl aliquots were washed three times in LB medium containing chloramphenicol (30 μg ml^-1^). For a comparable determination of nuclease activity in the respective culture supernatants, 230 μl aliquots of the same culture were used without washing in LB. Subsequently, cell suspensions were mixed with a 20 μl nucleic acid sample (833 bp PCR product) at a final concentration of 5 μg ml^-1^. The samples were incubated at 30°C, and 20 μl aliquots of each supernatant were removed, centrifuged, and analyzed by agarose gel electrophoresis at regular intervals.

#### DNA-Agar Assays

Appropriate *S. oneidensis* strains were inoculated from overnight cultures to an OD_600_ of 0.05 and incubated for 3 h while shaking. Cell suspensions were diluted 1:10 and 8 μl of the dilution were spotted carefully on DNA-agar plates (Beckton Dickinson GmbH, Heidelberg, Germany). After letting the spots dry, plates were incubated at RT for at least 48 h. 1N HCl was added carefully to plates to precipitate DNA. After incubating for 5 min plates were scanned using an Epson Perfection V700 Photo Scanner (Epson, Japan). Three independent assays were conducted.

#### GelRed Assays

Determination of the nuclease activity of purified ExeM, MBP-ExeM and its truncated versions was essentially performed as previously described (Heun et al., 2012). Briefly, in a 96-well plate 8 μg of enriched protein (corresponding to ∼25 pmol MBP-ExeM), 250 ng purified pBluescript II KS(+) DNA and 67 μl of 3× GelRed nucleic acid stain (Biotrend, Germany) were added to 10 mM Tris-HCl (pH 7.6) with 12.5 mM Mg^2+^ and Ca^2+^ in a final volume of 200 μl. Decrease of fluorescence of the GelRed stain were followed and recorded using a Tecan Infinite M200 microplate reader (Tecan, Switzerland). Most characterizations were carried out using amylose-enriched MBP-ExeM, which allowed using higher amounts of protein even though the amount of intact protein was lower (<50%). At least three independent assays were conducted.

### Membrane Separations

Inner and outer membrane fractions were separated and purified according to a sarkosyl-based protocol presented by Brown et al. (2010). *S. oneidensis* MR-1 was cultured overnight in 10 ml LB medium and reinoculated in 250 ml LB medium at an OD_600_ of 0.05. Strains with plasmid pBBMT-kan-*exeM* or derivatives (for the overproduction of ExeM or truncated ExeM variants) were cultured in the presence of 50 μg ml^-1^ kanamycin and induced with 0.2% (w/v) L-arabinose at an OD_600_ of 0.6. At an OD_600_ of 2, the cells were harvested by centrifugation at 10,000 *g* for 10 min. An 80 ml fraction of the culture was kept for isolation of the periplasmic fraction (see below). Unless stated otherwise, all centrifugations were performed at 4°C. The supernatant (SN) was ultracentrifuged twice at 35,000 *g* for 1 h and stored at -20°C for further analyzes. The cell pellet was suspended in 30 ml ice-cold 20 mM sodium phosphate buffer (pH 7.5) and passed one time through a prechilled “French press.” The clear lysate was centrifuged at 8,000 *g* for 10 min to remove unbroken cells, and an aliquot of the supernatant was stored as “whole cell lysate sample.” Ten milliliters of the remaining supernatant were ultracentrifuged at 45,000 *g* for 1 h. The supernatant was removed and centrifuged again to remove residual membrane fractions and insoluble protein and stored at -20°C as soluble fraction. The tube containing the whole membrane fraction was inverted to drain, and a sample was frozen in liquid nitrogen and stored at -20°C for further analyzes. The remaining whole membrane fraction was suspended in 0.5% sarkosysl (20 mM sodium phosphate) by frequent “pipetting” and orbital shaking at 220 rpm for 30 min at room temperature. The crude membrane suspension was ultracentrifuged at 45,000 rpm for 1 h to sediment the outer membrane. The supernatant containing the inner membrane was removed and the outer membrane sample was washed in ice-cold sodium phosphate buffer, spun down again by ultracentrifugation at 45,000 rpm for 1 h, suspended in 500 μl sodium phosphate, frozen in liquid nitrogen, and stored at -20°C. The supernatant containing the inner membrane was washed and concentrated to 500 μl using Vivaspin^®^6 centrifugation filter tubes (Sartorius Stedim Biotech GmbH, Germany) with a cutoff of 5 kDa. The inner membrane sample was frozen in liquid nitrogen and stored at -20°C for further analyzes.

The periplasmic protein fraction was isolated by osmotic shock according to Ross and coworkers (Ross et al., 2007). Eighty milliliters of the initial culture were centrifuged at 8,000 *g* for 10 min and suspended in 10 ml of 50 mM Tris-HCl, pH 8.0, 250 mM sucrose. The suspension was incubated for 5 min at room temperature and centrifuged at 8,000 *g* for 15 min. The pellet was suspended in ice-cold 5 mM MgSO_4_ and kept on ice with occasional inversion. The soluble periplasmic fraction was obtained from the supernatant after centrifugation at 8,000 *g* for 15 min. The periplasmic fraction was concentrated to a final volume of 500 μl using Vivaspin^®^6 centrifugation filter tubes (Sartorius Stedim Biotech GmbH, Germany) with a 5 kDa cutoff.

### Immunoblotting and Antibody Enrichment

Production of ExeM was determined using immunoblot analysis. Samples were taken from exponentially growing cultures and adjusted to an OD_600_ of 10. Immunoblot detection was essentially carried out as described earlier (Bubendorfer et al., 2012). Samples were separated by electrophoresis using 11% SDS-Polyacrylamide gels (SDS-PAGE) and subsequently transferred to a PVDF membrane by electroblotting. Detection of signals was carried out using the CDP-Star chemiluminescence substrate (Roche, Germany) and imaged using the FUSION-SL chemiluminescence imager (Peqlab, Erlangen, Germany).

The antibody against ExeM was ordered from Eurogentech (Germany) and further purified as follows. For purification of 2 ml of high-titer serum, 1 mg of ExeM was run on a gel and subsequently blotted to a PVDF membrane. The band of interest was cut and washed with acidic glycine buffer (100 mM Glycine, pH 2.5) for 5 min and then again washed twice with TBS [500 mM NaCl, 20 mM Tris, pH 7.4, 0.05% (v/v) Tween-20] for 2 min and blocked by soaking it with TBS-B (3% of fraction V bovine serum albumin in TBS buffer) for 1 h at RT while slightly shaking. The Membrane was washed twice with TBS and 2 ml of serum in 8 ml of TBS were added and incubated overnight at 4°C. Again, the membrane was washed twice with TBS and twice with PBS (0.135 NaCl, 3.5 mM KCl, 8 mM Na_2_HPO_4_, 2 mM KH_2_PO_4_, pH 7.4) each for 5 min. For elution 1 ml of acidic glycine buffer was added and after 10 min incubation at RT transferred to a new tube containing 1 M Tris, pH 8.0, bringing the final pH to 7.0. Elution was repeated and both fractions were pooled. Finally, antibodies were stored at 4°C with 5 mM sodium azide and 1 mg ml^-1^ of bovine serum albumin for stabilization. For detection, the antibodies were used in a 1:5,000 dilution. As a second antibody anti-rabbit coupled to goat AP was used in a 1:20,000 dilution. The antibody reliably detected purified MBP-ExeM and ExeM expressed in *S. oneidensis* MR-1 with only little unspecific binding to proteins in PAGE-separated crude extract (Supplementary Figure 1).

### Phylogenetic Analysis of ExeM-Like Nucleases

BLAST analysis was used to identify protein sequences showing high sequence similarities to ExeM (SO_1066) among the gammaproteobacteria. The sequences were aligned by ClustalW2 and subjected to phylogenetic analysis by PhylM using the LG substitution model and an aLRT-SH-like fast likelihood-based method (Larkin et al., 2007; Guindon et al., 2010). Phylogenetic trees were constructed by iTOL (Letunic and Bork, 2007). Branch lengths were disregarded for this analysis.

## Results

### ExeM Domain Structure

ExeM is a protein of 871 aa with a predicted molecular mass of 93.7 kDa. Protein domain analysis (**Figure 1**) by InterPro (Finn et al., 2017) suggest the presence of an N-terminal signal sequence (aa 1–24) followed by a lamin tail domain (LTD; aa 28–128) and a stretch (aa 28–128) resembling an oligonucleotide-binding structural motif similar to that of *Bacillus subtilis* YcgR, an endonuclease cleaving both RNA and DNA (Oussenko et al., 2004). A large domain belonging to the exonuclease-endonuclease-phosphatase (EEP) domain superfamily is predicted to account for almost the entire C-terminal half of the protein (aa 464–830). This EEP domain contains two residues (E521, D822) predicted to be involved in metal co-factor binding and three residues (H_642_, N_699_, H_823_), which are potentially involved in phosphate binding. The EEP domain is followed downstream by a prolin-rich stretch of 13 residues (aa 849–865; PAPVVPPKPQPTP) and a hydrophobic domain (aa 849–865; GGALGYLGLALLSLLGL) followed by four arginine residues (RRRR) at the very C-terminus of ExeM. This arrangement suggests that, while the major part of ExeM is excreted into the periplasm, the protein may remain anchored to the cytoplasmic membrane by a putative C-terminal transmembrane anchor the orientation of which is coordinated by the positively charged cytoplasmic four-arginine stretch (von Heijne, 1989, 1992; van de Vossenberg et al., 1998). Thus, release from the membrane may require proteolytic cleavage within the region linking the C-terminal membrane anchor and the EEP domain. Accordingly, the so-called “Gly-Gly-Cterm motif” consisting of a GG motif followed by a transmembrane helix and a cluster of basic residues has been predicted to constitute a processing signal for intramembrane serine proteases, referred to as rhombosortases. Accordingly, a putative rhombosortase (SO_2504) was also identified in *S. oneidensis* MR-1 (Haft and Varghese, 2011).

Potential orthologs to *So*ExeM with a highly similar domain structure were identified in other *Shewanella* and *Vibrio* species (such as *Vc*Xds), and also in species of *Aeromonas, Pseudoalteromonas* and, most distantly related, *Pseudomonas* (Supplementary Figure 2), which also lacks the putative C-terminal membrane anchor and rhombosortase processing site.

### *In Vitro* and *in vivo* Activity of ExeM

**FIGURE 2:**
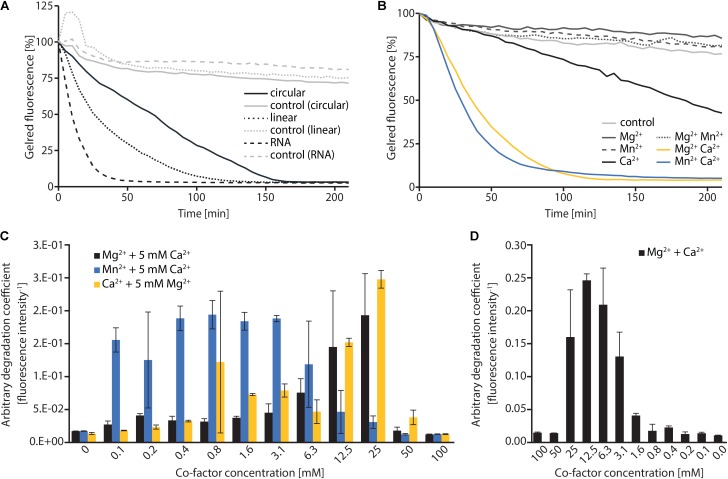
*In vitro* characterization of MBP-ExeM nucleolytic activity. **(A)** Degradation of pBluescript plasmid DNA (circular and linear; 250 ng) and RNA (1.8 μg) by MBP-ExeM (8 μg) as indicated by a loss in fluorescence of nucleic acid stain GelRed^TM^. The buffer contained 5 mM Mg^2+^ and Ca^2+^ to support ExeM’s nucleolytic activity. Control samples contained no MBP-ExeM. The assay was performed in triplicates in two independent experiments. The curves are based on the mean values of one representative experiment. **(B)** Degradation of pBluescript plasmid DNA (250 ng) by MBP-ExeM (8 μg; ∼25 pmol MBP-ExeM) as indicated by a loss in fluorescence of nucleic acid stain GelRed^TM^. The buffer contained 5 mM Mg^2+^, Mn^2+^, or Ca^2+^ (or combinations) to support ExeM’s nucleolytic actvity. The control samples contained no additional metal ions. The assay was performed in triplicates in two independent experiments. The curves are based on the mean values of one representative experiment. **(C)** Comparison of pBluescript plasmid DNA (250 ng) degradation by MBP-ExeM (8 μg protein; ∼25 pmol MBP-ExeM) in the presence of a range of concentrations of one cofactor, while the indicated second cofactor was kept at 5 mM. **(D)** Comparison of pBluescript plasmid DNA (250 ng) degradation by MBP-ExeM (8 μg) in the presence of Mg^2+^ and Ca^2+^ at different equimolar concentrations. The arbitrary degradation coefficient in **(C,D)** represents the reciprocal mean value of the fluorescence intensity (in % of the initial value) of nucleic acid stain GelRed^TM^ after 150 min (time point at which the fluorescence of at least one sample approached 0). Error bars represent standard deviations of two independent experiments performed each at least in triplicates.

To investigate the nucleolytic activity of ExeM, the role of the different domains and to identify potential co-factors, the protein was heterologously overproduced in *E. coli*. ExeM is predicted to be exported from the cytoplasm, and several bacterial nucleases have been shown to only fold into their active form after export into the periplasm to inhibit premature activity and to prevent loss of chromosome integrity. This can be conferred by disulfide bridge formation between cysteine residues (Wu et al., 2001; Li et al., 2003; Heun et al., 2012). Accordingly, four cysteine residues are present in ExeM (C_187_, C_272_, C_650_, C_670_; **Figure 1**) which may be involved in proper folding. Therefore, for heterologous production ExeM lacking its native signaling sequence as well as the C-terminal transmembrane region was targeted to the periplasm by an N-terminal fusion to maltose-binding protein (MBP) which also drastically increased yield and stability (Supplementary Figure 3). However, purified MBP-ExeM was still prone to rapid decay and aggregation which was further increased upon MBP-tag removal by TEV cleavage. Purified ExeM directly isolated from a gel subsequent to PAGE separation was used for antibody production, and for further *in vitro* assays MBP-ExeM was employed. In *in vitro* DNA-degradation assays ExeM readily degraded DNA and RNA as well as linear and plasmid DNA (**Figure 2A**) and therefore appears to act as a sugar-unspecific endonuclease. The presence of Ca^2+^ was crucial for enzyme activity and full activity additionally required Mg^2+^ or Mn^2+^ (**Figure 2B**). Other divalent cations (Zn^2+^, Ni^2+^, and Cu^2+^) did not affect nuclease function (data not shown). To determine the optimal concentration for these potential co-factors, a range of different concentrations was applied (**Figure 2C**). The results indicated that DNA degradation occurred most rapidly at concentration of 12–25 mM for Ca^2+^, 6–25 mM for Mg^2+^, and 0.1–6 mM for Mn^2+^. Highest activity of ExeM was found at an equimolar concentration of ∼12.5 mM for Ca^2+^ and Mg^2+^ (**Figure 2D**). Defining one unit of enzyme activity as the amount of MBP-ExeM required to completely degrade 1 μg of pBluescript vector within 10 min at 30°C in reaction buffer supplemented with 12.5 mM Mg^2+^ and 12.5 mM Ca^2+^, the specific activity of purified MBP-ExeM was ∼3 U/mg.

**FIGURE 3:**
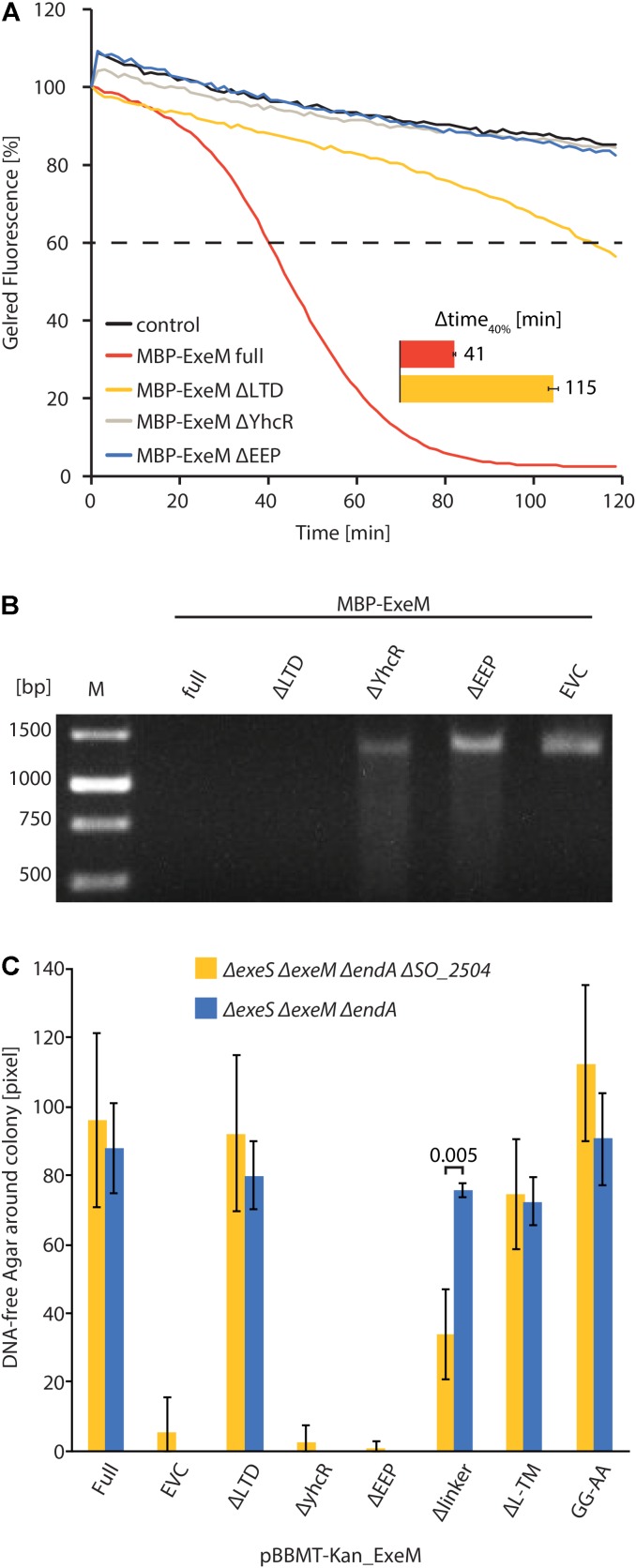
Influence of domain deletions of ExeM activity. **(A)** Degradation of 250 ng circular pBluescript plasmid DNA by different truncated versions of MBP-ExeM as indicated by a loss in fluorescence of nucleic acid stain GelRed^TM^. The buffer contained 5 mM Mg^2+^ and Ca^2+^ to support ExeM’s nucleolytic activity. Control samples contained no MBP-ExeM. The assay was performed in triplicates in three independent experiments. The curves are based on the mean values of one representative experiment. The inset (Δtime_40%_) indicates the time it took for 40% of the initial fluorescence to dissipate. **(B)** Degradation of a ∼1,400 bp PCR fragment by different truncated versions of MBP-ExeM over 1 h on a 1% agarose gel. M indicates GeneRuler^TM^ 1 kb DNA Ladder. **(C)** Degradation of high molecular weight DNA in agar plates displayed as DNA free radius around colonies overexpressing different ExeM versions in pixels. The indicated values are the mean of three independent experiments. Error bars represent the standard deviation. Only for one mutant version of ExeM (Δlinker) a significant difference was seen in the absence of the putative rhombosortase (SO_2504), as indicated by the *p*-value. The significance threshold was adjusted from *p* < 0.05 to *p* < 0.00625 using *Bonferroni correction*. *p*-Values are given in Supplementary Table 1.

To further determine the role of the putative ExeM domains we heterologously overproduced versions in which one of the major predicted domains was deleted (MBP-ExeM_ΔLTD_; MBP-ExeM_ΔYcgR_; MBP-ExeM_ΔEEP_) and applied these for *in vitro*-testing (**Figure 3A**). Loss of the YcgR-like and the EEP domains resulted in a complete inactivity of the nuclease. The ExeM version truncated by the N-terminal LTD domain showed slow but significant DNA degradation in the fluorescence-based assay, and a visible DNA fragment was absent after agarose separation after incubation with ExeM_ΔLTD_ (**Figure 3B**).

To test the *in vivo* activity of ExeM and corresponding mutant versions, the appropriate *S. oneidensis* strains were incubated on DNA agar and the extracellular nucleolytic activity was determined as a measure of clearance of turbidity around the colonies due to DNA degradation (**Figure 3C**). Comparison of the clearance area revealed that loss of ExeM resulted in a significant decrease in extracellular degradation, and, accordingly, approximately wild-type levels of extracellular DNA degradation were observed when ExeM was the only remaining nuclease. To determine the activity of the mutated versions of the protein, *exeM* and the corresponding variants (*exeM*_ΔLTD_; *exeM*_ΔYcgR_; *exeM*_ΔEEP_) were expressed from a plasmid under control of an inducible promoter. To avoid interference with the activity of the native extracellular nucleases, EndA, ExeM, ExeS, the expression was carried out in a strain in which all three nucleases were deleted (*ΔendA ΔexeM ΔexeS*). The strain expressing wild-type *exeM* displayed the expected zone of DNA clearance while mutants lacking the *ycgR*-like and the EEP domains were inactive. In contrast, expression of *exeM*_ΔLTD_ resulted in a clearing zone of almost the size of wild-type *exeM*. Thus, the YcgR-like and the EEP domain are crucial for ExeM function and/or stability while the LTD domain is dispensible for extracellular DNA degradation.

**FIGURE 4:**
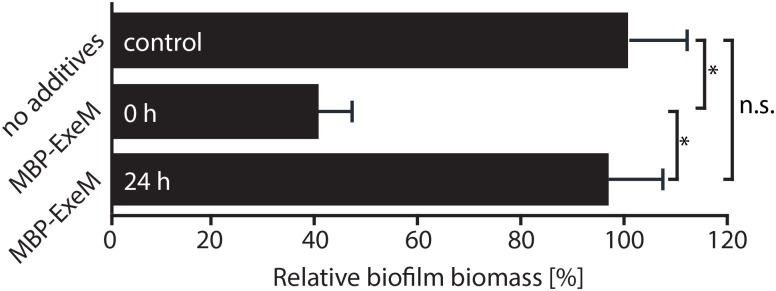
Effect of exogenously added MBP-ExeM on biofilm formation. Biofilm formation (static conditions) of the *Shewanella oneidensis* MR-1 wild-type after addition of MBP-ExeM (0.025 U) compared to an untreated control culture. MBP-ExeM was added prior to inoculation (0 h) or after 24 h of incubation (24 h; and incubated for further 2 h after addition). The values are means of three replicates. Error bars represent standard deviations. Asterisks indicate a significant difference between the two samples as calculated by standard *t*-tests after *Bonferroni correction* (*p* < 0.017). *p*-values are 1.5 × 10^-12^ (comparing control and 0 h), 0.43 (control and 24 h), and 2.5 × 10^-09^ (24 and 0 h), respectively.

Previous studies indicated that ExeM is implicated in *S. oneidensis* MR-1 biofilm formation and benefits biofilm formation under static conditions (Gödeke et al., 2011a; Heun et al., 2012). We therefore determined the effect of externally added ExeM on biofilm formation (**Figure 4**). We found that, when purified MBP-ExeM was added to attaching cells, biofilm formation was significantly diminished. In contrast, 24 h-old biofilms were no more affected as no significant release of biomass could be detected upon addition of ExeM (**Figure 4**). This may suggest that ExeM may negatively affect biofilm formation when expressed in incorrect amounts or already in attaching cells.

### Transport, Localization, and Processing of ExeM

The domain structure of ExeM strongly indicates that the protein is transported across the cytoplasmic membrane but may remain tethered by the C-terminal transmembrane domain and requires proteolytic cleavage, e.g., by a rhombosortase, to be released. Earlier proteome studies have identified ExeM within the cell envelope, however, it is unclear whether the nuclease is associated within the inner or outer membrane (Tang et al., 2007; Brown et al., 2010). Earlier studies suggested weak activity of ExeM in the medium supernatant (Gödeke et al., 2011a). Therefore, ExeM transport across the cell envelope and the nuclease’s final destination remained controversial.

**FIGURE 5:**
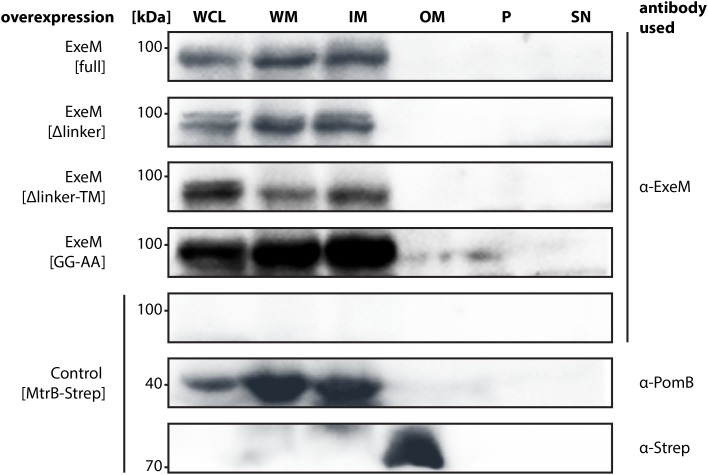
Localization of full-length ExeM and truncated ExeM constructs in various cellular fractions of *S. oneidensis* MR-1. Immunodetection of ExeM (α-ExeM antibody), PomB (α-PomB antibody), and MtrB-Strep (α-Strep antibody) in the whole cell lysate (WCL), the whole membrane fraction (WM), the inner membrane fraction (IM), the outer membrane fraction (OM), the periplasmic fraction (P), and the cell-free culture supernatant (SN). Full-length ExeM and truncated variants were overproduced in the *ΔendA ΔexeS ΔexeM* nuclease mutant. The *ΔendA ΔexeS ΔexeM* nuclease mutant overproducing MtrB-Strep was utilized as control for outer-membrane location and PomB as a control for inner-membrane location.

To gain a better understanding of the ExeM localization and transport, three mutants of ExeM were produced: ExeM_Δlinker_, lacking the region between the EEP and the putative C-terminal transmembrane domain, ExeM_Δlinker-TM_, lacking both C-terminal linker and transmembrane domain, and ExeM_GG-AA_, in which the putative rhombosortase processing site (GG) was substituted (for an overview see **Figure 1**). The corresponding genes *exeM* and the various mutant versions were expressed at low levels from a plasmid under control of an arabinose-inducible promoter as the native production levels turned out to be too low to be reliably detected by the polyclonal antibody raised against purified ExeM. To prevent possible functional interference with the natively expressed extracellular nucleases in *S. oneidensis* MR-1, the mutant strain *S. oneidensis* MR-1 *ΔendA ΔexeS ΔexeM* was used for production. The strains were grown in planktonic culture to exponential growth phase, and the whole cell extract, the outer and inner membrane fractions of the cells were prepared. In addition, the periplasmic and supernatant fractions were harvested and concentrated. Subsequently, the presence of ExeM and the corresponding mutant variations were determined by immunoblot analysis of the different fractions (**Figure 5**).

ExeM was almost exclusively detected in the fractions containing the inner membrane, strongly indicating that the nuclease is at least transiently associated with the inner membrane. Notably, this was also observed for some amount of ExeM lacking the C-terminal transmembrane region, which potentially represented ExeM in the process of being transported or associated with the cytoplasmic membrane also in the absence of the C-terminal domain. Substitution of the putative rhombosortase processing site (ExeM_GG-AA_) resulted in an increase of ExeM abundance in the inner membrane, suggesting that cleavage from the inner membrane may occur. In addition, ExeM variants lacking the linker region occurred as double bands, also indicating processing. However, no ExeM in significant amounts could be detected in the outer membrane fraction but also not within the periplasmic fraction or the supernatant. As earlier experiments have indicated potential ExeM activity in cell free abstracts, these experiments were repeated under conditions of phosphate starvation, which have been shown to increase *exeM* expression (Gödeke et al., 2011a). Also the analysis of lyophilized supernatant of these cultures did not identify any amounts of ExeM (data not shown). The results suggest that under planktonic conditions at least the major part of ExeM remains associated with the inner membrane after export and may be rather unstable upon release.

To further determine whether rhombosortase-mediated processing of ExeM is required for activity under conditions of surface growth, we measured the zone of clearance of ExeM producing strains on DNA-agar plates (**Figure 3C**). To this end, ExeM was produced in a strain lacking the three extracellular nucleases (*ΔendA ΔexeS ΔexeM*) and also in a strain additionally lacking the putative rhombosortase SO_2504 (*ΔendA ΔexeS ΔexeM ΔSO_2504*). The results clearly demonstrated that, generally, processing by SO_2504 is not required for the extracellular nucleolytic activity of ExeM as the zone of clearance was only little affected in the absence of the putative rhombosortase, and also the GG to AA substitution of the predicted cleaving site motif had little effect. In addition, deletion of the C-terminal transmembrane anchor decreased the extracellular ExeM degradation activity by some 20%, indicating that release of ExeM from the inner membrane does not increase its activity. Thus, processing by a rhombosortase or other proteases is not necessarily required for ExeM function under these conditions. However, in a strain producing an ExeM variant lacking the linker region displayed a pronounced decrease in extracellular nucleolytic activity, suggesting that this linker region affects activity or stability of ExeM.

## Discussion

In previous studies, we assigned the primary function of ExeM in *S. oneidensis* MR-1 to biofilm formation (Gödeke et al., 2011a; Heun et al., 2012). However, molecular features that determine functional specificity in *S. oneidensis* MR-1 biofilm formation are yet to be elucidated. In particular, some characteristics inherent by ExeM that are required for modulation, processing, and degradation of biofilm-specific eDNA as well as ExeM activation and transport remain elusive. The results obtained in this study represent a first basis for elucidating ExeM’s structure–function relationships and its role in biofilm formation of *S. oneidensis* MR-1. As the nuclease ExeM is conserved in *Shewanellaceae, Vibrionaceae, Aeromonadaceae*, and *Pseudoalteromonadacae*, we expect our findings to, similarly, extent to other bacterial species.

Our *in vitro* degradation assays with purified MBP-ExeM fusion protein strongly indicated that ExeM is a sugar-unspecific endonuclease, as linear and circular DNA as well as RNA substrates were readily degraded. Notably, previous studies on Xds, the nuclease ortholog to ExeM in *V. cholerae*, implicated that Xds functions as an exonuclease (Seper et al., 2011). The Xds data were generated using culture supernatants containing Xds instead of enriched protein in *in vitro* assays, which may explain these functional differences. Protein homology analyzes suggested that ExeM belongs to the diverse superfamiliy of two-metal-ion-dependent nucleases which also include DNaseI (Yang, 2011), and putative metal-binding sites were identified within the EEP domain by *in silico* analysis (**Figure 1**). Accordingly, we found that ExeM strictly requires Ca^2+^ as co-factor, and full activity occurred in the presence of Mg^2+^ but also Mn^2+^⋅ Ca^2+^ and Mg^2+^ are highly common co-factors for nucleases (Yang, 2011), and also Mn^2+^ has already been identified previously as functional co-factors for nucleases such as for *B. subtilis* YcgR as well as for another *S. oneidensis* extracellular endonuclease, EndA (Oussenko et al., 2004; Heun et al., 2012). *Shewanella* sp. are well-adapted to redox-stratified environments and are capable of releasing soluble Mn^2+^ ions from highly abundant, but rather insoluble manganese minerals by respiratory electron transfer (Hau and Gralnick, 2007). Thus, increased local Mn^2+^ concentrations can occur in appropriate environments and were found to occur in Lake Oneida sediments from which *S. oneidensis* MR-1 was originally isolated (Myers and Nealson, 1988). However, whether this finding holds any physiological relevance with respect to an environmental adaption of *Shewanella* sp. or rather is an *in vitro* effect due to similar properties of Mg^2+^ and Mn^2+^ cations is so far unclear.

All three major predicted putative domains (LTD, YcgR-like, EEP) are required for full ExeM activity, however, a truncated version lacking the N-terminal LTD domain still displayed residual activity. LTDs are found in eukaryotic nuclear lamins and several uncharacterized proteins from phylogenetically diverse bacteria and some archaea. In bacteria these domains mainly occur with membrane-associated hydrolases of the metallo-β-lactamase, synaptojanin, calcineurin-like phosphoesterase superfamilies, or in secreted or periplasmic proteins associated with oligosaccharide-binding domains or as multiple tandem repeats in a single protein (Mans et al., 2004; Dittmer and Misteli, 2011). The role of LTD domains has not yet been elucidated, but according to the general predicted function of the prokaryotic proteins harboring LTD domains, a potential role in directing proteins to the membrane or membrane-associated structures was suggested (Mans et al., 2004). Our results are not inconsistent with such a role of the LTD domain in ExeM. However, as ExeM-LTD *in vitro* function in membrane-free environments is decreased, the LTD domain may also be directly involved in the nucleolytic activity. In contrast to the LTD domain, the presence of the YcgR-like region is crucial for ExeM function as the deletion results in an almost complete loss of nucleolytic activity. The YcgR-like region comprises two putative OB-fold nucleic acid–binding regions and may therefore be involved in substrate interaction (Theobald et al., 2003; Oussenko et al., 2004).

Two previous proteomic studies on *S. oneidensis* MR-1 have identified ExeM to be associated with the cell envelope. However, it remained unclear whether the nuclease was localized to the cytoplasmic (Brown et al., 2010) or the outer membrane (Tang et al., 2007). In our study, ExeM almost exclusively occurred in the cytoplasmic membrane fraction, independently of production levels. As *in vivo* assays using DNA plates showed that active ExeM was produced upon ectopic *exeM* expression, we concluded that this localization pattern was unlikely to be exclusively due to potential effects of the overproduction of a secreted protein and also corresponded to the presence of the putative C-terminal membrane anchor. This and the presence of a putative rhombosortase processing site suggested that ExeM may be released from the membrane upon appropriate proteolytic processing. However, we found that the absence of the rhombosortase or substitutions in the predicted cleavage site did not have a major effect on membrane association or ExeM-mediated DNA-degradation on DNA-agar plates. An exception to this occurred in rhombosortase-free mutants expressing an ExeM variant solely lacking the linker region connecting the protein with the predicted C-terminal membrane anchor. In this case, the extracellular nucleolytic activity was diminished to about 40% of wild-type levels. This may be explained by the finding, that in *S. oneidensis* MR-1, the rhombosortase is predicted to process a number of extracellular proteases which, in the absence of cleavage, may remain and accumulate in the cytoplasmic membrane (Haft and Varghese, 2011). This may affect the stability of linker-lacking ExeM if the protein remains close to the cytoplasmic membrane.

In addition, association of ExeM with the inner membrane still occurred in the absence of the predicted membrane anchor, and, furthermore, although earlier studies suggested that some ExeM-mediated activity may occur in culture supernatants (Gödeke et al., 2011a), this finding could not be verified in the absence of the other two *S. oneidensis* MR-1 extracellular nucleases, ExeS and, in particular, EndA (Heun et al., 2012) (this study). Accordingly, we did not detect ExeM in highly concentrated medium supernatants even under phosphate-limiting conditions or when overproduced. Similar results were reported from the proteome analysis of *S. oneidensis* MR-1 culture supernatants grown under phosphate-limiting conditions in a chemostat (Pinchuk et al., 2008). It may therefore be hypothesized that ExeM remains associated with the cytoplasmic membrane and only small amounts of this nuclease are released, which may also be mediated by cell lysis rather than active transport. It may also be speculated that DNA is transported into the periplasm to be degraded by ExeM, as potential orthologs to DNA import systems, e.g., to that of *V. cholerae* (Matthey and Blokesch, 2016) can readily be identified also in *Shewanella* sp. Thus, ExeM may have an additional role in restricting the entry of extracellular DNA into the cells.

Like for many other bacterial species, DNA is a major structural compound of *S. oneidensis* MR-1 biofilms and assemblages (Pinchuk et al., 2008; Gödeke et al., 2011b; Binnenkade et al., 2014). Under hydrodynamic conditions, loss of ExeM results in the formation of biofilms with tightly packed cells, which contain elevated amounts of extracellular DNA and in which biofilm dispersal is highly reduced (Gödeke et al., 2011a; Heun et al., 2012). In contrast, the presence of ExeM is required for normal biofilm formation of *S. oneidensis* under static conditions, and in its absence, biofilm formation is significantly decreased (Gödeke et al., 2011a). This is not intuitive, as an accumulation of extracellular DNA would rather be expected to lead to more robust surface–attached communities also under these conditions. In this study we found that externally added purified MBP-ExeM negatively affected biofilm formation but was unable to disperse already formed biofilms. We therefore hypothesize that correct timing and amount of ExeM production is crucial for function in biofilms. Since ExeM was predominantly present in the cell envelope and could not be detected in supernatants, we further hypothesize that ExeM may act on extracellular DNA which is in close proximity to or directly interacting with the cell envelope. ExeM may therefore act at the individual cell level to promote or fine–tune proper cell–cell interactions or detachment. It can also not be excluded that ExeM is involved in supporting biofilms cells with phosphate, although in *S. oneidensis* this role appears to be rather fulfilled by the nuclease EndA (Heun et al., 2012). Clarification of the role ExeM plays in *S. oneidensis* MR-1 biofilm formation therefore requires further studies. In addition, the ExeM ortholog of *V. cholerae*, Xds, was demonstrated to be involved in degrading the structurally crucial DNA-scaffold of eukaryotic NETs, thus lowering the susceptibility for NET-mediated extracellular killing (Seper et al., 2013). If such a role of ExeM also applies to *Shewanella* sp., in particular *S. algae* and *S. putrefaciens* which have been identified as commensal pathogens (Janda and Abbott, 2014), remains to be shown.

## Author Contributions

LB, MK, and KT conceived experiments, discussed results, and wrote the manuscript. LB and MK conducted experiments.

## Conflict of Interest Statement

The authors declare that the research was conducted in the absence of any commercial or financial relationships that could be construed as a potential conflict of interest.
