# Supplementary material for: Characterization of ExeM, an Extracellular Nuclease of Shewanella oneidensis MR-1
Source: Front Microbiol. 2018 Aug 3;9:1761. doi: 10.3389/fmicb.2018.01761 (PMC6085458; doi:10.3389/fmicb.2018.01761)
Supplement: Supplementary file 1 [file Data_Sheet_1.PDF]

Supplementary Material

Characterization of ExeM, a multifunctional extracellular nuclease of *Shewanella oneidensis* MR-1

Lucas Binnenkade[1,#], Maximilian Kreienbaum[1,#], Kai M. Thormann[1,*]

[1]Institute for Microbiology and Molecular Biology, Justus-Liebig-Universität Giessen, D-35392 Giessen, Germany

*** Correspondence:**
Kai Thormann
kai.thormann@mikro.bio.uni-giessen.de

[#] **equal contribution**

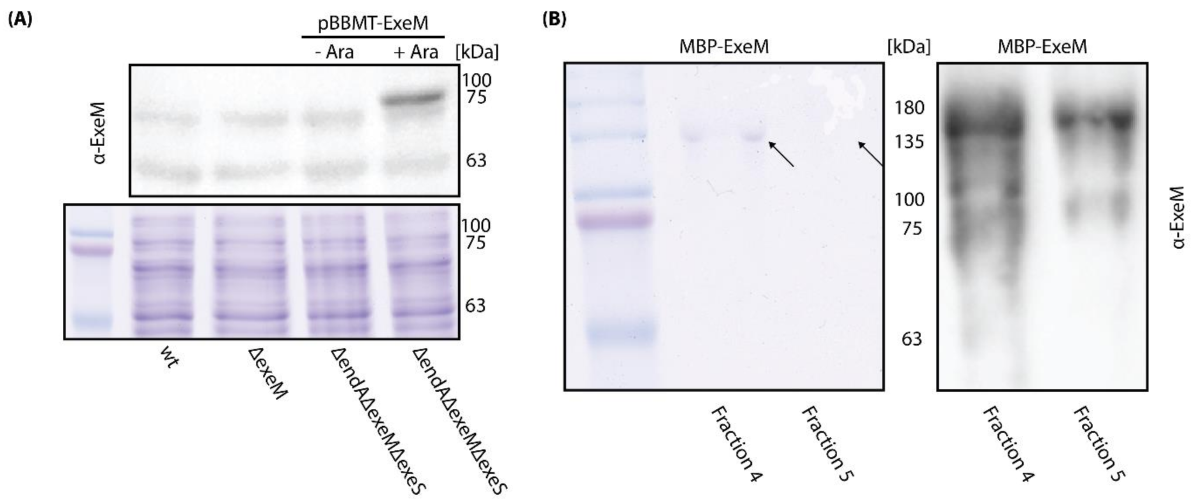

Supplemental Figure 1. Immunoblot analysis of ExeM. (A) Coomassie-stained SDS-PAGE and western blot of whole cell samples of wild-type *S. oneidensis* MR-1, *exeM* deletion mutant and a mutant lacking all three extracellular nucleases. The latter harbors the *exeM* overexpression plasmid pBBMT-ExeM, which is induced by adding 0.2 % arabinose. **(B)** Coomassie-stained SDS-PAGE and Western blot of the two main elution fractions after gel filtration column.

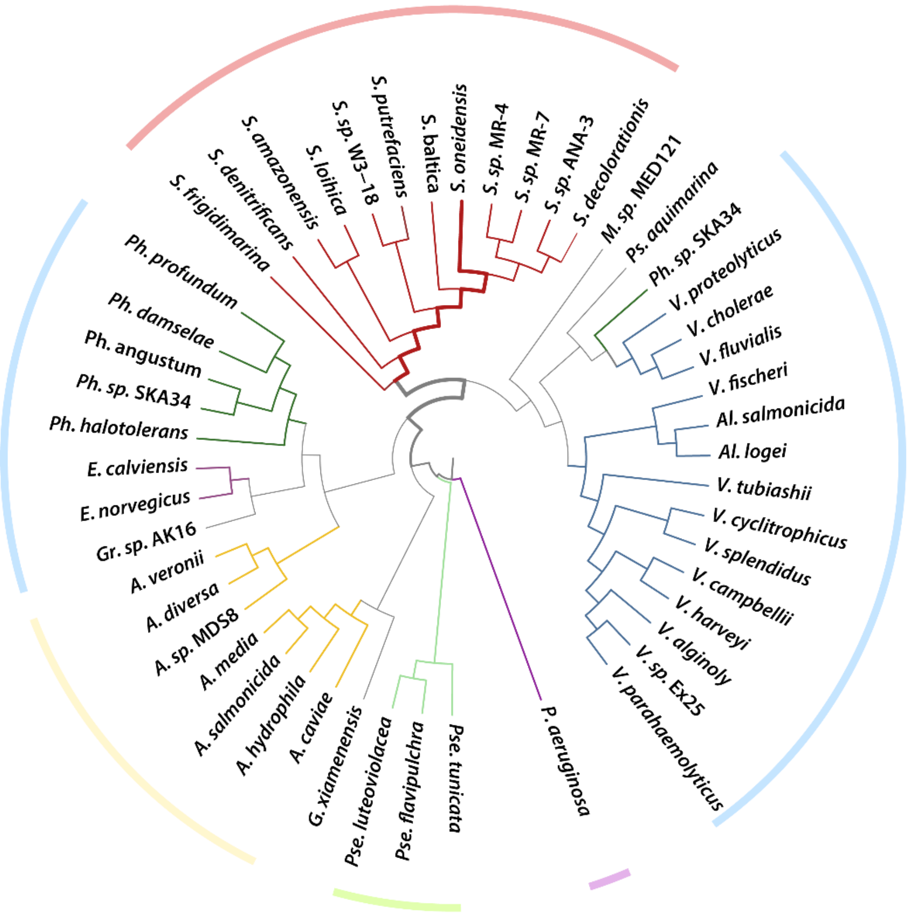

Supplemental Figure 2. Phylogenetic analysis of ExeM-like nucleases. BLAST analysis was used to identify protein sequences showing high sequence similarities to ExeM (SO_1066) among the gammaproteobacteria. The sequences were aligned by ClustalW2 and subjected to phylogenetic analysis by PhylM using the LG substitution model and an aLRT-SH-like fast likelihood-based method (Larkin et al. 2007, Guindon et al. 2010). Phylogenetic trees were constructed by iTOL (Letunic and Bork 2007). Branch lengths were disregarded for this analysis. Branch colors highlight identical genera. Genera abbreviations: S., *Shewanella*; M., *Marinomonas*; Ps., *Psychromonas*; Ph., *Photobacterium*, V., *Vibrio*; P., *Pseudomonas*; Pse., *Pseudoalteromonas*; G. *Gallaecimonas*; A. *Aeromonas*; Gr., *Grimontia*; E., *Enterovibrio*. Circle colors indicate taxonomic classification of the individual species to one family: red, *Shewanellaceae*; blue, *Vibrionaceae*; purple, *Pseudomonadaceae*; green, *Pseudoalteromonadaceae*; yellow *Aeromonadaceae*.

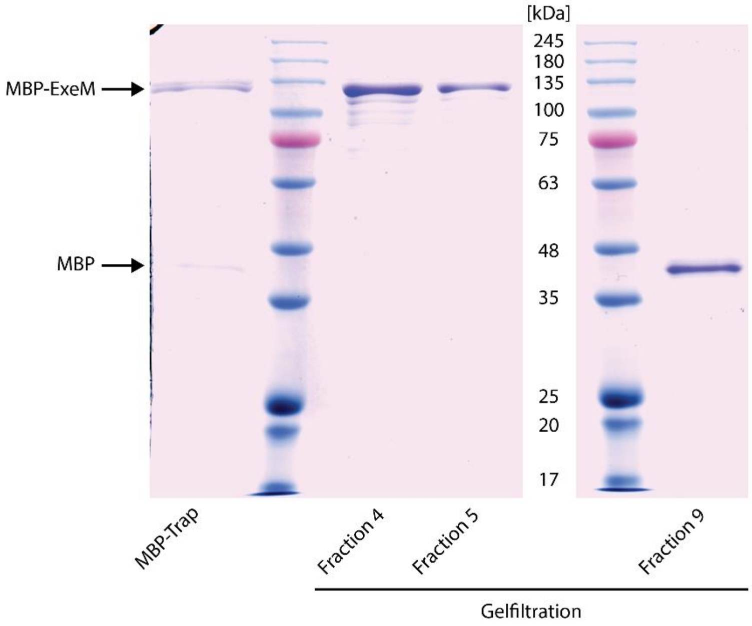

Supplemental Figure 3. SDS-PAGE of purified MBP-ExeM. Coomassie-stained SDS-PAGE of the overexpression and purification of MBP-ExeM. Depicted are the main fractions of the MBP-Trap and the gel filtration columns. Fraction 4 and 5 of the gel filtration harbor the vast majority of MBP-ExeM, while fraction 9 through 11 (data not shown) contain unspecifically cleaved of MBP.

Supplemental Table 1. Statistical significance of differences between the *ΔexeSΔexeMΔendA* mutant strain and a mutant strain additionally lacking SO_2504 when overexpressing different versions of ExeM (see Fig. 3C). *p*-values were obtained by performing standard t-tests for pairwise comparison and are based on three independent experiments. Bonferroni correction was used to adjust the significance threshold from $p < 0.05$ to $p < 0.00625$.

**ExeM version**	Full	EVC	ΔLTD	ΔyhcR	ΔEEP	Δlinker	ΔL-TM	GG-AA
**p-Value**	0.651	0.374	0.446	0.374	0.374	0.005	0.827	0.225

Supplemental Table 2. Bacterial strains that were used in this study

Strain	relevant genotype	reference or source
Escherichia coli		
BL-21 Star (DE3)	F^- *ompT hsdS*$_B$(r_B^- m_B^-) *gal dcm rne131* (DE3)	Invitrogen (Thermo Fisher Scientific), USA
DH5α λ pir	Φ80*dlacZ*ΔM15 Δ(*lacZYA-argF*) U196 *recA1 hsdR17 deoR thi-l supE44 gyrA96 relA1/ λpir*	Miller and Mekalanos 1988
WM3064	*thrB*1004 *pro thi rpsL hsdS lacZ*ΔM15 RP4-1360 Δ(*araBAD*)567 Δ*dapA*1341::[*erm pir*(wt)]	W. Metcalf, Univ. of Illinois, Urbana
Shewanella oneidensis		
MR-1	*S. oneidensis* MR-1 wild type	
S988	*ΔexeM*	Gödeke, Heun et al. 2011
S989	*ΔexeS*	Gödeke, Heun et al. 2011
S1034	*ΔexeM ΔexeS*	Gödeke, Heun et al. 2011
S2095	*ΔendA*	Heun, Binnenkade et al. 2012
S2096	*ΔexeM ΔendA*	this work
S2097	*ΔexeS ΔendA*	this work
S2160	*ΔexeM ΔexeS ΔendA*	this work
S3191	*ΔLTD-exeM*	this work
S3195	*ΔyhcR-exeM*	this work
S3199	*ΔEEP-exeM*	this work
S3203	*ΔL-TM-exeM*	this work
S3207	*Δlinker-exeM*	this work
S3211	*GG-AA-exeM*	this work
S3187	*ΔSO_2504*	this work
S3920	*ΔexeM ΔexeS ΔendA ΔSO_2504*	this work
S4195	*ΔgspD*	this work
S4196	*ΔgspD ΔexeS ΔendA*	this work
S4197	*ΔgspD ΔexeS ΔendA ΔexeM*	this work

Supplemental Table 3. Plasmids that were used in this study.

Plasmid	purpose	reference or source
pBAD-mtrB-Strep	Construct for overproduction of Strep-tagged MtrB (SO_1776) in *Shewanella oneidensis* MR-1	J. Gescher, University of Freiburg
pBBMT-kan-exeM	Wild type gene copy of exeM and RBS in pBBMT-kan for overproduction in *Shewanella oneidensis* MR-1	this work
pBBMT-kan-exeM-GG-AA	exeM construct encoding G849A and G850A aa substitutions in putative linker region + RBS in pBBMT-kan for overproduction in *Shewanella oneidensis* MR-1	this work
pBBMT-kan-exeM-ΔEEP	Deletion of putative EEP domain in exeM (encoding aa 464-830) + RBS in pBBMT-kan for overproduction in *Shewanella oneidensis* MR-1	this work
pBBMT-kan-exeM-ΔLTD	Deletion of putative LTD domain in exeM (encoding aa 28-128) + RBS in pBBMT-kan for overproduction in *Shewanella oneidensis* MR-1	this work
pBBMT-kan-exeM-ΔL-TM	Deletion of putative linker region in exeM (encoding aa 833-865) + RBS in pBBMT-kan for overproduction in *Shewanella oneidensis* MR-1	this work
pBBMT-kan-exeM-ΔYhcR	Deletion of putative YhcR domain in exeM (encoding aa 220-291) + RBS in pBBMT-kan for overproduction in *Shewanella oneidensis* MR-1	this work
pMal-P2-TEV	Overexpression vector, N-teminal MBP fusion, TEV protease cleavage site, periplasmic secretion	this work
pMal-P2-TEV-ExeM	Encoding ExeM without N-terminal signal peptide and C-terminal transmembrane region (aa 27-849) in pMAL-P2-TEV	this work
pMal-P2-TEV-ExeM-ΔEEP	Encoding ExeM without N-terminal signal peptide and C-terminal transmembrane region (aa 27-849) and the EEP domain in pMAL-P2-TEV	this work
pMal-P2-TEV-ExeM-ΔLTD	Encoding ExeM without N-terminal signal peptide and C-terminal transmembrane region (aa 27-849) and the LTD domain in pMAL-P2-TEV	this work
pMal-P2-TEV-ExeM-ΔyhcR	Encoding ExeM without N-terminal signal peptide and C-terminal transmembrane region (aa 27-849) and the yhcR domain in pMAL-P2-TEV	this work

Supplemental Table 4. Oligonucleotides that were used to create the strains and plasmids used in this study.

name	sequence	purpose
MO11 d0833-PstI-up-fw	CGACTGCAGGCTGTAACTCCATCGCTGG	In-frame deletion of SO_0833
MO12 d0833-up-overlap-rev	CGCCAACAATTAGCCGAATAAGGTCGACAATG	In-frame deletion of SO_0833
MO13 d0833-dwn-overlap-fw	TAATTGTTGGCGGTTCAACATTTATCCCAAAAAT AATC	In-frame deletion of SO_0833
MO14 d0833-dwn-BamH1-rev	AGGATCCGCATTTGCAGCAAAATCGT	In-frame deletion of SO_0833
MO15 d0833-check-fw	CGCGCGTATGGGATAAAT	Check deletion of SO_0833
MO16 d0833-check-rev	CCAACCACTCAATTTACCCTTAG	Check deletion of SO_0833
O28 dSO1844_up_EcoRI_fw	CGAATTCCGAGCGGGGAGAGTTATTACT	In-frame deletion of SO_1844
O29 dSO1844_up_overlap_rv	CGTGGTATCCGCCAACAGTGTTTTGTGTT	In-frame deletion of SO_1844
O30 dSO1844_dwn_overlap_fw	CTGTTGGCGGATACCACGACACTGACATATCGT	In-frame deletion of SO_1844
O31 dSO1844_dwn_PstI_rev	CGACTGCAGCTGCAAAATCGGTAGTTTTTTCA	In-frame deletion of SO_1844
O32 dSO1066_up_EcoRI_fw	CGAATTCTCAGTAAACCTAGTCATATTAAGGG	In-frame deletion of SO_1066
O33 dSO1066_up_overlap_rv	TTGTAAACCAGAAACAGCTGTTAACTTATTAAC A	In-frame deletion of SO_1066
O34 dSO1066_dwn_overlap_fw	GCTGTTTCTGGTTTACAACGTCGTCGC	In-frame deletion of SO_1066
O35 dSO1066_dwn_PstI_rev	CGACTGCAGCCCATTTGCACTATTACCG	In-frame deletion of SO_1066
O36 dSO1844_check_fw	GGTCAAGCAGAACACTTCAGC	Check deletion of SO_1844
O37 dSO1844_check_rev	GCTTCTCTACTGTAGGGGATCC	Check deletion of SO_1844
O38 dSO1066 _check_fw	GGTATTTTTTTATGCGATTGAATCC	Check deletion of SO_1066
O39 dSO1066 _check_rev	GGATCACCACACTTGGCATC	Check deletion of SO_1066
P271 TEV-pMAL-Fw	GAAAACCTGTATTTTCAGGGCATTTCAGAATTCG GATCCTCTAGAG	Creation of pMal-P2-TEV
P272 no_Xa-pMAL-Rev	CCCGAGGTTGTTGTTATTGTTATTG	Creation of pMal-P2-TEV
P273 9xHis-no_Xa-pMAL-Rev	GTGATGATGGTGATGGTGGTGATGATGCCCGAG GTTGTTGTTATTGTTATTG	Creation of pMal-P2-TEV
P274 Seq-pMAL-TEV-Fw	TGAAGCCCTGAAAGACGCGC	Creation of pMal-P2-TEV

P330 PspOMI-US-dSO_2504-Fw	ACTGGGCCCCTCAACGAATTAGCCATTTCTGCC	In-frame deletion of SO_2504
P331 OL-US-dSO_2504-Rev	TTTACGCTTTCAGTTTCACGCCTGTGGCTTACC	In-frame deletion of SO_2504
P332 OL-DS-dSO_2504-Fw	CGTGAAACTGAAAGCGTAAATACTTGATTCTTCTTTGATG	In-frame deletion of SO_2504
P333 EcoRI-US-dSO_2504-Rev	TCTATGAATTCGCGTAACTTGGAGTTTGAACAGG	In-frame deletion of SO_2504
P334 chk-dSO_2504-Fw	CGAAATCCCTCGCCCAACGC	Check deletion of SO_2504
P335 chk-dSO_2504-Rev	GCGCAGCTTAGCCATGATATCG	Check deletion of SO_2504
MK128 KO_gspD_US_fw	GCGAATTCGTGGATCCAGATCATTACCAATGCCGGTCGCT	In-frame deletion of *gspD*
MK129 KO_gspD_US_rv	TTCACTCATTGTGTTTTATCCCGTTTGTTATTCATTATTTTTCTTTCC	In-frame deletion of *gspD*
MK130 KO_gspD_DS_fw	AAATAATGAATAACAAACGGGATAAAACACAATGAGTGAAA	In-frame deletion of *gspD*
MK131 KO_gspD_DS_rv	GCCAAGCTTCTCTGCAGGATGGTTTCGATATGAATATCCG	In-frame deletion of *gspD*
MK146 chk_KO_gspD_fw	GCCAAGAAACCTACAGCTTG	Check deletion of *gspD*
MK147 chk_KO_gspD_rv	GCGACACCAATAAAGATGAC	Check deletion of *gspD*
P353 NheI-RBS-exeM-pBBMT-Fw	ACTGCTAGCAGGAGGGCAAATATGGAAAATGTTAATAAGTTAACAGCTGTTTC	Creation of pBBMT carrying different ExeM versions
P354 PspOMI-exeM-pBBMT-Rev	ACTGGGCCCTCAATAACGGCGACGACGTTGTAAACC	Creation of pBBMT carrying different ExeM versions
P238 BamHI_exeM_noSP_Fw	ATAGGATCCATGATCACTGAGTATGTTGAAGGTAGC	Creation of pMal-P2-TEV-ExeM variants
P239 SalI_exeM_noTM_Rev	CTAGTCGACTTACTTTGGCGTCGGCTGCGGTTTTGG	Creation of pMal-P2-TEV-ExeM variants
MK122 SalI_OE_exeM_LTD_fw	TTTCAGAATTCGGATCCATGAATGTAATGGACGTGCCAACCCC	Creation of pMal-P2-TEV-ExeM variants
MK123 SalI_OE_exeM_rv	GCAGGTCGACTCTAGAGTTACTTTGGCGTCGGCTGCGGTT	Creation of pMal-P2-TEV-ExeM variants
MK124 SalI_OE_exeM_notLTD_fw	TTTCAGAATTCGGATCCATGAATGTAATGATCACTGAGTATGTTGAAGGTAGCTCA	Creation of pMal-P2-TEV-ExeM variants

References

Miller, V. L. and J. J. Mekalanos (1988). "A novel suicide vector and its use in construction of insertion mutations: osmoregulation of outer membrane proteins and virulence determinants in *Vibrio cholerae* requires toxR." J Bacteriol 170:2575–2583.

Venkateswaran, K., et al. (1999). "Polyphasic taxonomy of the genus *Shewanella* and description of *Shewanella oneidensis* sp. nov." Int J Syst Bacteriol 49:705–724.

Gödeke, J., Heun, M., Bubendorfer, S., Paul, K. and K. M. Thormann (2011). "Roles of two *Shewanella oneidensis* MR-1 extracellular endonucleases." Appl Environ Microbiol 77:5342–5351.

Heun, M., L. Binnenkade, M. Kreienbaum and K. M. Thormann (2012). "Functional specificity of extracellular nucleases of *Shewanella oneidensis* MR-1." Appl Environ Microbiol 78(12): 4400-4411.
